# Return to Physical Activity After High Tibial Osteotomy or Unicompartmental Knee Arthroplasty: A Systematic Review and Pooling Data Analysis

**DOI:** 10.1177/0363546520948861

**Published:** 2020-09-22

**Authors:** James Belsey, Sam K. Yasen, Simon Jobson, James Faulkner, Adrian J. Wilson

**Affiliations:** † University of Winchester, Winchester, UK; ‡ Hampshire Hospitals NHS Foundation Trust, Basingstoke, UK; § The Wellington Hospital, London, UK; Investigation performed at Department of Sport, Exercise and Health, University of Winchester, Winchester, UK

**Keywords:** high tibial osteotomy, unicompartmental knee arthroplasty, unicondylar knee arthroplasty, physical activity, return to sport, outcome, quality of life, indications, knee replacement

## Abstract

**Background:**

The 2 most common definitive surgical interventions currently performed for the treatment of medial osteoarthritis of the knee are medial opening wedge high tibial osteotomy (HTO) and medial unicompartmental knee arthroplasty (UKA). Research exists to suggest that physically active patients may be suitably indicated for either procedure despite HTO being historically indicated in active patients and UKA being more appropriate for sedentary individuals.

**Purpose:**

To help consolidate the current indications for both procedures regarding physical activity and to ensure that they are based on the best information presently available.

**Study Design:**

Systematic review.

**Methods:**

A search of the literature via the MEDLINE, Embase, and PubMed databases was conducted independently by 2 reviewers in accordance with the PRISMA (Preferred Reporting Items for Systematic Meta-Analyses) guidelines. Studies that reported patient physical activity levels with the Tegner activity score were eligible for inclusion. Patient demographics, operative variables, and patient-reported outcome scores were abstracted from the included studies.

**Results:**

Thirteen eligible studies were included, consisting of 401 knees that received HTO (399 patients) and 1622 that received UKA (1400 patients). The patients’ mean age at surgery was 48.4 years for the HTO group and 60.6 years for the UKA group. Mean follow-up was 46.6 months (HTO) and 53.4 months (UKA). All outcome scores demonstrated an equal or improved score for activity and knee function regardless of the operation performed. Operative variables during HTO had a larger effect on outcome than during UKA.

**Conclusion:**

Patients who underwent HTO were more physically active pre- and postoperatively, but patients undergoing UKA experienced an overall greater increase in their physical activity levels and knee function according to Tegner and Lysholm scores. Activity after HTO may be influenced by operative factors such as the implant used and the decision to include a graft material in the osteotomy gap, although this requires further research. Some studies found that patients were able to return to physical activity postoperatively despite having an age or body mass index that would traditionally be a relative contraindication for HTO or UKA.

The 2 most common definitive surgical interventions currently performed for the treatment of medial osteoarthritis (OA) of the knee are medial opening wedge (OW) high tibial osteotomy (HTO) and medial unicompartmental knee arthroplasty (UKA). The traditional indications for HTO include unicompartmental OA, tibial deformity, no extreme knee instability, >120° range of motion, age <60 years, physically active, and body mass index (BMI) <30 kg/m^2^.^1,7,60^ The traditional indications for UKA include unicompartmental OA, age >60 years, angular deformity <15°, low functional demands, and body mass <82 kg.^14,15,60^ However, a wide body of research exists to suggest that good outcomes can be achieved with either procedure well outside these traditional indications. Specifically, physically active patients may be suitably indicated for either procedure.^10,14^

Surgeons have historically favored HTO when presented with physically active patients and opted for UKA in cases of more sedentary individuals.^50^ A recent study, however, showed that patients who underwent UKA for medial OA participated in higher levels of postoperative physical activity after 3 months and 2 years as compared with those who underwent HTO.^22^ While it is well-reported that patients are able to return to physical activity postoperatively in most cases of HTO,^12^ 2 recent reviews found the same to be true for patients undergoing UKA.^54,56^ With more studies emerging that report positive results after UKA where the traditional indications regarding physically active patients have not been adhered to, a comparative overview of the current situation around return to physical activity after HTO and UKA would be timely. Such an analysis would allow for the review and consolidation of the current indications for both procedures to ensure that they are based on the best information presently available. Ultimately, this would serve to improve surgical patient selection to the benefit of future patients. Notwithstanding the aforementioned advantages of a review focused on return to physical activity after surgery, to our knowledge recent systematic reviews and meta-analyses comparing HTO and UKA have focused on issues such as survivorship/revision, pain, complications, and knee function but have not focused sufficiently on return to physical activity.^8,14,15,17,26,28,43,50^

The implementation of patient-reported outcome questionnaires is common to assess the outcome of HTO and UKA, and the Tegner activity scale is one such questionnaire that is often used to assess patient physical activity levels after either procedure.^12,54^ The Tegner activity score is based on a 10-point scale where 0 represents a patient who is on sick leave from work as a result of knee problems, 5 represents a job involving heavy labor or participation in activities such as competitive cycling or recreational jogging on uneven ground, and 10 represents a patient who plays competitive high-impact sports such as soccer at the national or international level.^52^

The purpose of the present study was to perform a systematic review of the literature to investigate patients’ return to physical activity after HTO or UKA.

## Methods

**Figure 1.**
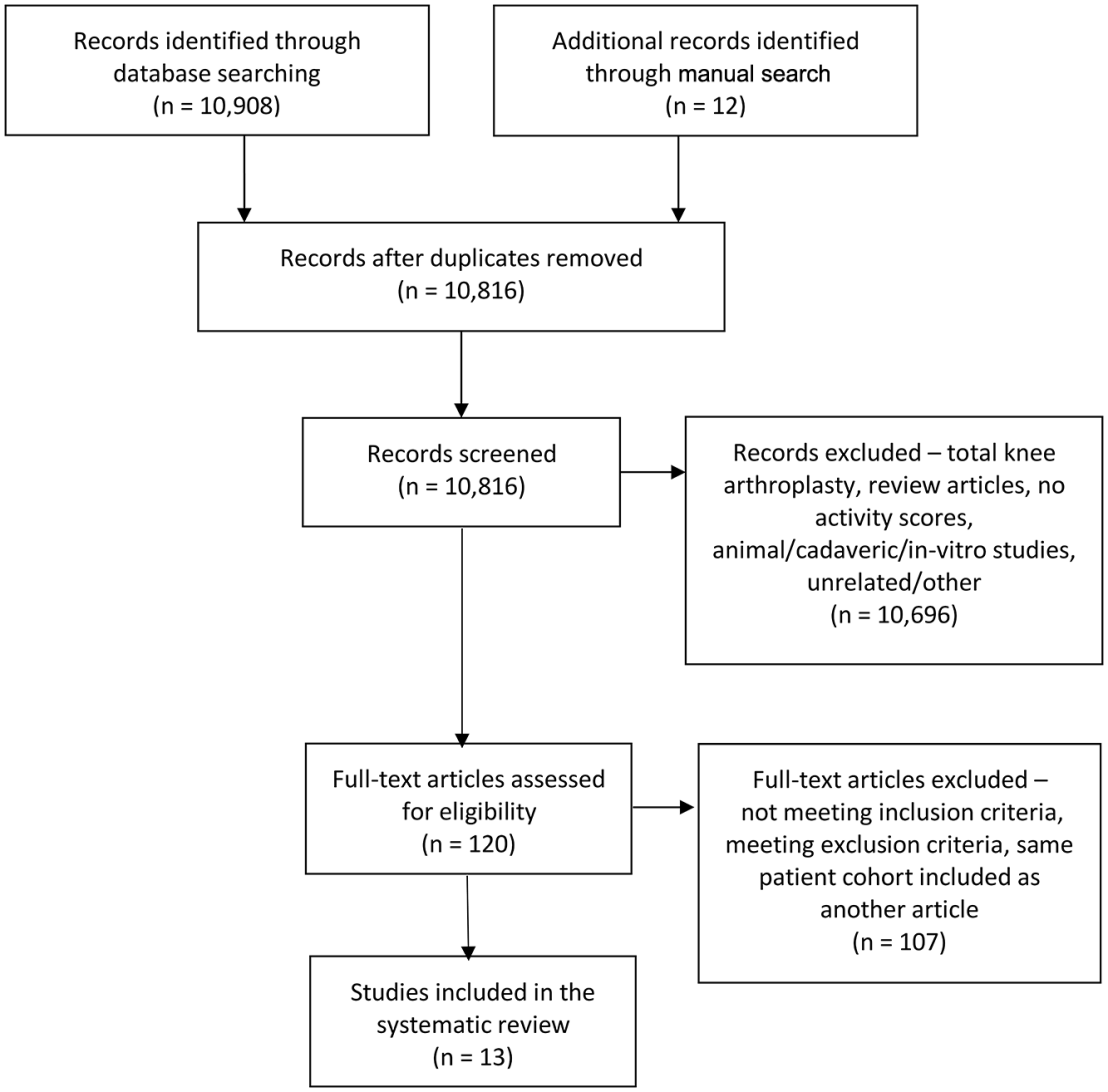
Search results flowchart following the PRISMA (Preferred Reporting Items for Systematic Meta-Analyses) guidelines.

A search of the literature with the MEDLINE, Embase, and PubMed databases was conducted independently by 2 authors (J.B., S.K.Y.) following the PRISMA (Preferred Reporting Items for Systematic Reviews and Meta-Analyses) guidelines (Figure 1).^29,47^ Basic and Medical Subject Headings (MeSH) searches were performed within each database; the search terms for which can be found in Table 1. Articles were then screened and assessed for eligibility for inclusion in the review by 2 authors (J.B., S.K.Y.) according to the following criteria: in vivo study with human participants, full text in English, internal plate fixation (for HTO), medial OW HTO, medial UKA, and Tegner activity scale scores reported. Additionally, articles were excluded from the review per the following criteria: sample included revision surgery, patients with anterior cruciate ligament deficiency, use of a novel surgical technique (defined as being unique and experimental at the time of publication), and an unspecified type of osteotomy or arthroplasty. The reference lists of any previous reviews and meta-analyses were also manually searched to identify any additional published studies for inclusion. Unpublished studies and conference abstracts were not included.

**Table 1:** Basic and MeSH Search Terms Used^*a*^

Basic Search Terms	MeSH Search Terms
1. UKR	1. Knee arthroplasty [MeSH]
2. UKA	2. Knee replacement [MeSH]
3. Unicompartmental knee replacement	3. Arthroplasty, replacement, knee [MeSH]
4. Unicompartmental knee arthroplast*	4. (1 OR 2 OR 3)
5. Unicondylar knee replacement	5. Tibia osteotomy [MeSH]
6. Unicondylar knee arthroplast*	6. Osteotomy [MeSH]
7. Partial knee replacement	7. (5 OR 6)
8. Partial knee arthroplast*	8. Physical activity, capacity and performance [MeSH]
9. (1 OR 2 OR 3 OR 4 OR 5 OR 6 OR 7 OR 8)	9. Return to Sport [MeSH]
10. Tibia* osteotom*	10. Exercise [MeSH]
11. Knee osteotom*	11. (8 OR 9 OR 10)
12. HTO	12. (4 AND 11)
13. (10 OR 11 OR 12)	13. (7 AND 11)
14. Sport*	
15. Phys* activ*	
16. (14 OR 15)	
17. (9 AND 16)	
18. (13 AND 16)	

a Asterisk denotes truncated term. MeSH, Medical Subject Headings.

### Methodological Quality Assessment

The methodological quality of each included article was assessed with the Methodological Index for Non-Randomized Studies (MINORS), a 12-point checklist that has been validated for use with nonrandomized studies (comparative and noncomparative). Each item on the checklist was given a score between 0 and 2, where 0 means that the item was not reported in the article; 1 signifies that the item was reported in the article but was “inadequate”; and 2 denotes that the item was reported and was “adequate.”^49^ The ideal global score for noncomparative studies was calculated with 8 items on the MINORS checklist, meaning that a maximum score of 16 was possible. All 12 items on the checklist were used to calculate a score for comparative studies, meaning that an ideal global score of 24 was possible. A study with high methodological quality was defined as one that satisfied at least 50% of the criteria.^53^ Nine articles included in the final review were noncomparative studies and had a mean ± SD MINORS score of 11 ± 0.9. Two articles compared HTO against UKA^22,57^ and had a mean MINORS score of 19 ± 1.4. The comparative and noncomparative studies had, on average, “fair” methodological quality.^23^

An additional 2 articles included in the present systematic review were randomized controlled trials (RCTs).^32,35^ The methodological quality of these studies was assessed by comparing the articles against the revised CONSORT (Consolidated Standards of Reporting Trials) statement, a 22-point checklist designed to guide authors of RCTs when writing up their findings to improve their reports^30^; the higher the score, the better the methodological quality of a given study (Table 2).

**Table 2:** Articles Included in Systematic Review (n = 13)^*a*^

				Knees at	Male:				Tegner		Methodological
Author (Year)	Study Type	Technique	Implant	Follow-up	Female	Age, y	BMI, kg/m^2^	Follow-up, mo	Pre	Post	Quality
Bastard (2017)^3^	Retrospective cohort	Medial OW HTO + synthetic graft	Limmed locking plate	30	6:24	55.6 (27-59)	33.52 (22.9-41.6)	16 (12-18)	4 (3-6)	4 (3-6)	12/16 (MINORS)
Faschingbauer (2015)^13^	Retrospective cohort	Medial OW HTO + no graft	Tomofix	43	32:11	42 ± 11.2	26.9 ± 3.6	22 ± 9.3	3.78 ± 1.9	3.7 ± 1.4	10/16 (MINORS)
Jahnke (2014)^18^	Prospective cohort	Medial UKA	Oxford	147	72:63	63.5 (36-86)	Not reported	24 ± 17.6	4.06 ± 1.4	3.9 ± 0.96	12/16 (MINORS)
Krych (2017)^22,*b*^	Prospective comparative	Medial OW HTO	Not reported	39	29:10	41	31.2	86	3.1 ± 1.4	3.3 ± 1.2	20/24 (MINORS)
		Medial UKA	Miller-Galante fixed bearing	183	82:101	49.2	32.4	70	2.6 ± 0.9	4.5 ± 0.9	
Nerhus (2017)^32^	Prospective RCT	Medial OW HTO	Puddu plate	35	20:15	51.3 (34-59)	Not reported	24	2.2 (2-3)	2.9(2.4-3.3)	18/22 (CONSORT)
Pandit (2011)^34^	Prospective cohort	MI medial UKA; cemented	Oxford phase III	547	393:425	66 (32-88}	Not reported	60 (12-132)	2.3 ± 1.1	2.8 ± 1.1	11/16 (MINORS)
Pandit (2013)^35^	Prospective RCT	MI medial UKA; cementless	Oxford phase III	27	16:14	64.7 (45-82)	27.9 (21-40)	60	1.9 ± 0.7	2.9 ± 0.6	19/22 (CONSORT)
		MI medial UKA; cemented	Oxford phase III	32	20:12	63.8 (46-78)	28.9 (20-38)	60	1.9 ± 0.8	2.6 ± 0.8	
Panzram (2018)^36^	Retrospective cohort	Medial UKA; cementless	Oxford phase III	27	15:12	62.5 (49-76)	Not reported	60 (47-69)	2.9 ± 1.4	3.4 ± 1.0	9/16 (MINORS)
Salzmann (2009)^42^	Retrospective cohort	Medial OW HTO + no graft	Tomofix	65	51:14	41.2 ± 5.6 (19-65)	27.1 ± 3.7 (20-34)	36 ± 8.1 (14-84)	4.9 ± 2.3 (1-10)	4.3 ± 1.5 (2-9)	11/16 (MINORS)
Saragaglia (2014)^44^	Prospective cohort	Medial OW HTO	Not reported	62	39:23	50.5 ± 10.3	27.06 ± 4.6	69 ± 15.6 (60-108)	4.6 ± 1.7	4.2 ± 1.4	11/16 (MINORS)
Schröter (2013)^46^	Retrospective cohort	Medial OWHTO + autograft	Limited contact dynamic compression plate	32	22:10	47 ± 9.0	28.6 ± 4.7	77 ± 19.0	3.0 ± 1.4	4.1 ± 1.3	11/16 (MINORS)
Walker (2015)^55^	Prospective cohort	MI medial UKA; cemented	Oxford phase III	109	46:47	55 (36-60)	32 (20-58)	53 ± 19.0 (28-101)	2.0 ± 1.1 (1-6)	3.8 ± 1.1	11/16 (MINORS)
Yim (2013)^57,*b*^	Retrospective comparative	Medial OW HTO (+ allograft chips if gap >10 mm)	Two wedge plates	58	7:51	58.3 ± 5.4 (43-65)	Not reported	43 ± 5.0 (36-48)	3.1 ± 1.1	2.5 ± 1.2	18/24 (MINORS)
		Medial UKA	Miller-Galante fixed bearing	50	2:48	60.3 ± 4.5 (47-65)	Not reported	44 ± 5.0 (36-48)	3.2 ± 0.9	2.6 ± 0.9	

a Values are presented as No. or mean ± SD (range). BMI, body mass index; CONSORT, Consolidated Standards of Reporting Trials; CW, closing wedge; HTO, high tibial osteotomy; MI, minimally invasive; MINORS, Methodological Index for Non-Randomized Studies; OW, opening wedge; Post, postoperative; Pre, preoperative; RCT, randomized controlled trial; UKA, unicompartmental knee arthroplasty.

b Study comparing HTO and UKA groups.

### Data Abstraction and Analysis

The following data were extracted and recorded from each study: author, year of publication, study type, operation type (HTO or UKA), operative technique, implant type, sample size, mean age at surgery, sex, BMI, mean follow-up, and mean pre- and postoperative outcome scores. In all studies except 1, by Pandit et al,^34^ where postoperative outcomes were reported at multiple time intervals,^22,32,35^ the most recent postoperative interval was included in the review. The study by Pandit et al^34^ reported postoperative outcomes at 1, 5, 7, and 10 years. It was noted in the article that only 156 of the original 1000 operated knees (a loss to follow-up of 84%) provided outcome scores at 10 years. Given the overall loss to follow-up (28%) and mean final follow-up (4.1 years) of the other studies included in the present review, the 547 knees that had outcome scores at 5 years in the Pandit et al^34^ study were included in the final synthesis of data to reduce the effects of attrition bias and skewed data.

Three studies reported the data of different HTO techniques: medial OW, lateral closing wedge (CW),^22,32^ or double^44^ osteotomy. The study by Nerhus et al^32^ included separate data sets for the demographics and outcome scores of its OW and CW cohorts; only the data of the OW group were included in the present review. It was not possible to separate the medial OW HTO data in the articles by Saragaglia et al^44^ and Krych et al.^22^ The authors were contacted and asked to provide this information, which was then included in the final review. Schröter et al^46^ reported only median Tegner scores. As such, the lead author was contacted, and the mean values were obtained. A final HTO study met the inclusion criteria but reported only the mean change in pre- to postoperative Tegner scores, rather than separately stating the baseline and follow-up values.^20^ As a result, this study was excluded from the overall review because of the unavailability of the required data.

A noncomparative UKA study met the inclusion criteria, but the sample combined 3 lateral UKA cases with 25 medial UKA cases.^45^ It was not possible to extract the data relating to patients who underwent medial UKA. This study was therefore excluded from the review. One RCT compared cementless versus cemented fixation during medial UKA,^35^ but no differences were found between the methods preoperatively or at final follow-up. Hence, this study was suitable for inclusion, and the demographic and outcome data from both groups were included in the final review.

## Results

### Literature Search

The titles and abstracts of the 10,908 studies resulting from the database searches and the 12 studies from the manual search were first screened for duplicates. Additionally, any articles that did not qualify for the present study depending on the inclusion and exclusion criteria were removed at this stage based on their title and abstract.

The full texts of the remaining 120 articles were again screened according to the inclusion and exclusion criteria. A subset of patients constituting 74% of the overall cohort in 1 study^25^ was part of a larger cohort of patients in 2 other articles.^16,34^ As such, this study was excluded from the final review. The study by Hamilton et al^16^ did not include preoperative Tegner scores and included only postoperative scores for various subsets of their cohort. This study was excluded from the final review. The study by Pandit et al^34^ did include pre- and postoperative Tegner scores and was therefore included to represent this patient cohort in the final review. Pandit et al^33^ included the same sample of patients as a subsequent report by the same lead author^35^; as such, the earlier article was excluded and the more recent article included. Details about the 13 studies included in the final systematic review can be found in Table 2.

A total of 2097 knees (1873 patients) were eligible for inclusion in the 13 studies. Of this total, 74 knees (74 patients) underwent CW HTO or double osteotomy and were excluded from the present review. Of the remaining participants, 696 knees were lost to follow-up, resulting in scores from 1327 knees being pooled and reviewed. It was not possible to report the total number of patients representing the 1327 knees at final follow-up, as this was not reported in the study by Pandit et al,^34^ which accounted for 547 knees (40% of the overall sample). All studies included in the present review met the minimum requirement for methodological quality. The 9 noncomparative studies scored a mean 10.9 of 16 (range, 9-12), and the 2 comparative studies scored 18 of 24 and 20 of 24 according to the MINORS criteria.^22,57^ The 2 RCT reports scored 18 of 22 and 19 of 22 according to the CONSORT statement.^32,35^

### Operative Technique

**Figure 2.**
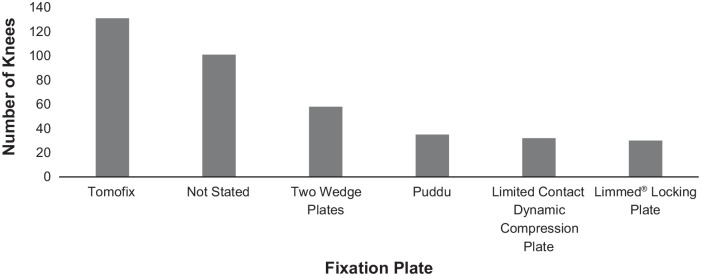
Total number and type of medial opening wedge high tibial osteotomy internal fixation plates used in the included studies.

**Figure 3.**
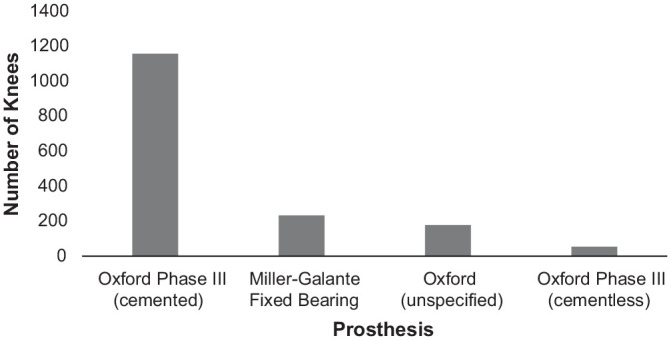
Total number and type of unicompartmental knee arthroplasty prostheses used in the included studies.

A total of 401 knees (399 patients) underwent medial OW HTO, and 1622 knees (1,400 patients) underwent medial UKA. Bone grafting was used in 62 HTO knees^3,46^ and in an unspecified number of knees in the study by Yim et al.^57^ Figures 2 and 3 show the pooled types of HTO fixation plates and UKA prostheses used in the studies. The Tomofix plate was the most common HTO fixation plate, as used in 32.7% of the included sample (131 knees). Type of internal plate fixation was not reported in 2 studies,^22,44^ which constituted 25.2% of the total sample (101 knees). The remaining HTO studies used different fixator plates. With respect to UKA, in 71.3% of the sample (1157 knees), a cemented Oxford phase III prosthesis was used. The 178 knees (159 patients) in the study by Jahnke et al^18^ also received an Oxford UKA, but it was not clear whether this was a phase III prosthesis. It is therefore possible that the overall percentage of patients who received a phase III prosthesis was >71.3%.

### Demographics

The mean age at surgery for all patients was 54.5 years, and 857 male and 875 female patients were recruited for these studies (excluding CW HTO and double osteotomies). The male:female ratio of patients who were lost to follow-up is not known. When all patients were categorized by operation, the mean age at surgery for the HTO and UKA groups was 48.4 and 60.6 years, respectively. Additionally, where reported, more men underwent HTO than women (211:153), while the inverse was true for patients who underwent UKA (646:722). Of the 9 studies that reported BMI, mean overall BMI was 29.56 kg/m^2^ (29.06 kg/m^2^ for HTO and 30.30 kg/m^2^ for UKA). The mean overall follow-up was 50.3 months (46.6 months for HTO and 53.4 months for UKA).

### Patient-Reported Outcome Scores

Outcome scores at final follow-up were available for 322 HTO knees and 1005 UKA knees, representing a loss to follow-up of 34% (20%, HTO; 38%, UKA). In addition to the Tegner scores, other patient-reported questionnaires were used to accrue more clinical outcome data. The 3 most common questionnaires were the Lysholm score, Oxford Knee Score, and the University of California, Los Angeles (UCLA), activity scale. The Lysholm score is a subjective measure of patients’ day-to-day knee function and general condition.^6^ Lysholm scores were reported for 322 HTO knees and 116 UKA knees in 6 HTO studies^3,13,32,42,44,46^ and 2 HTO/UKA comparative studies.^22,57^ The Oxford Knee Score, which is designed to assess the outcome of knee surgery,^31^ was applied in 1 HTO study^32^ and 3 UKA studies^18,34,35^ representing 35 HTO knees and 753 UKA knees. Similar to the Tegner score, the UCLA activity scale determines participation levels in various physical activities.^58^ Two HTO studies^32,44^ and 3 UKA studies^18,36,55^ reported UCLA scores, which corresponded to 97 HTO knees and 283 UKA knees. Table 3 shows the pooled mean reported pre- and postoperative levels for these clinical outcome scores. All scores demonstrated an equal or improved score for activity and knee function regardless of the operation performed.

**Table 3:** Mean Clinical Outcome Scores^*a*^

	Tegner		UCLA		Lysholm		OKS	
	Pre	Post	Pre	Post	Pre	Post	Pre	Post
HTO	3.6	3.6	6.3	6.3	57.8	76.6	26.3	36.7
UKA	2.6	3.3	4.8	6.4	65.5	90.2	25.5	35.0
Overall	3.1	3.5	5.4	6.4	59.5	79.3	25.7	35.3

a HTO, high tibial osteotomy; OKS, Oxford Knee Score; Post, postoperative; Pre, preoperative; UCLA, University of California, Los Angeles, activity scale; UKA, unicompartmental knee arthroplasty.

## Discussion

The HTO group reported higher activity levels pre- and postoperatively than the UKA group, who in turn exhibited greater overall pre- to postoperative improvement in physical activity according to the Tegner scores. Pooled analysis of the most commonly used outcome scores in the included studies showed that patients who underwent UKA demonstrated greater improvement in their knee conditions according to the Lysholm scores but that knee function according to the Oxford Knee Score was similar between procedures. The pooled UCLA scores largely supported the pooled Tegner scores by showing that the HTO group was more physically active preoperatively than the UKA group and that a similar level of activity was maintained postoperatively. Additionally, patients undergoing UKA exhibited a larger pre- to postoperative increase in physical activity. These findings demonstrate the propensity for HTO to be used in more active patients and UKA to be performed in patients who are preoperatively more sedentary.

The minimal clinically important difference (MCID) for the Tegner score was previously estimated at 0.85,^20,22^ which was not achieved pre- to postoperatively in either group. The MCID of the pre- to postoperative changes in the Lysholm score (9.9 points) and Oxford Knee Score (5 points) was achieved in both groups.^9,20,22^ The MCID of the UCLA score is not known. The mean preoperative Tegner scores demonstrated that patients undergoing HTO were involved in light-moderate labor, competitive low-impact sports such as swimming, and recreational high-impact sports such as cross-country skiing or jogging on even ground. In comparison, mean preoperative Tegner scores for the UKA group were equivalent to light labor and walking on uneven ground. Mean postoperative Tegner scores for both groups were similar to the mean preoperative scores of the HTO group. A more highly active HTO patient group preoperatively supports the traditional indications for both procedures with regard to patient activity levels and suggests that they are being adhered to in most cases.

Other traditional indications for HTO, such as younger age and BMI <30 kg/m^2^, were reflected in the present review—specifically, the HTO group was 12.2 years younger than the UKA group and had a mean BMI of 29.1 kg/m^2^. However, patients in 3 of the studies included in this review were not consistent with these indications.^3,22,55^ The study by Walker et al^55^ specifically investigated patients aged <60 years who underwent UKA. Mean Tegner and UCLA scores improved significantly from 2.0 and 3.3 to 3.8 and 6.8, respectively, at a mean 53-month follow-up. Similarly, the study by Krych et al,^22^ which included patients undergoing UKA at a mean age of 49.2 years, demonstrated an overall mean improvement in Tegner scores from 2.6 preoperatively to 4.5 at a mean 70-month follow-up. The improvement in physical activity levels reported by Walker et al and Krych et al suggests that age may not be a limiting factor with regard to return to physical activity after UKA. However, HTO has been shown to be more cost-effective than UKA in patients aged <60 years.^5,21^

Furthermore, if patients undergo UKA at a younger age than what is traditionally indicated, then attention must be paid to the endpoint of such procedures and their effect on subsequent revision to total knee arthroplasty (TKA). A previous meta-analysis showed that revision to TKA after UKA occurred 8.2 years after surgery, whereas revision to TKA after HTO occurred 9.7 years after surgery.^50^ Two review articles, including 1 meta-analysis, suggested that revising UKA to TKA led to worse outcomes as compared with primary TKA.^48,51^ Conversely, the literature tends to suggest that this is not the case when revising HTO to TKA.^10,38,53^ Additionally, Robertsson and W-Dahl^40^ found that TKA after UKA had an increased risk of subsequent revision as compared with TKA after HTO. High revision rates of UKA to TKA have also been shown in the United Kingdom’s National Joint Registry, which records the outcomes of >100,000 partial and total knee arthroplasties performed annually, leading to results based on very large sample sizes that support the previously mentioned literature.^39^ There is limited evidence to suggest that UKA performs well in the short to midterm in patients younger than the traditional indication for this procedure.^22,55^ However, the higher cost of UKA versus HTO, the shorter time until revision to TKA as compared with HTO, the worse outcomes of TKA, and the increased risk of subsequent revision of TKA as reported in the literature suggest that caution should be exercised when UKA is offered to patients aged <60 years.

In addition to age, the traditional BMI range for patients indicated for HTO has not always been strictly adhered to. In the HTO study by Bastard et al,^3^ the mean BMI of patients was 33.5 kg/m^2^. Despite being higher than the traditionally recommended BMI threshold for HTO, patients had equaled their preoperative levels of physical activity at a mean 16-month follow-up according to the Tegner scores. This was consistent with the pooled analysis of the other HTO studies in the present review.

The operative technique during HTO and UKA has many associated variables that could have an effect on outcome, which was a major contributing factor to the heterogeneity of the reviewed studies. When Tegner data were pooled for the 2 HTO studies (62 knees) reporting that the osteotomy gap was filled with graft material,^3,46^ a pre- to postoperative improvement in physical activity from 3.5 to 4.1 was observed. The 5 HTO studies (202 knees) that did not fill the osteotomy gap reported no change in physical activity levels, with pre- and postoperative Tegner scores equaling 3.7.^13,22,32,42,44^ These findings suggest that the inclusion of a graft during HTO may affect the outcome and could allow for a return to physical activity at a level higher than that preoperatively, although further investigation is required to confirm this.

Overall, 3 HTO studies^22,32,46^ showed a postoperative increase in physical activity, while 1 study^3^ demonstrated no change and the remaining 4 showed a decrease in physical activity levels according to the Tegner scores. Conversely, 5 UKA studies^22,34,35,36,55^ in the review showed a postoperative increase in physical activity according to the Tegner scores, while 2 studies^18,57^ documented a decrease. The variation in HTO results as compared with the consensus reached among most UKA studies suggests that UKA may lead to a more predictable increase in physical activity than HTO, although patients who underwent HTO remained more active overall. However, it might equally demonstrate that the outcome of HTO is more sensitive to the surgical technique employed and equipment used than is the case with UKA. Given the variation of results presented in the literature, these findings make evident the need for further investigation into return to physical activity after surgery, particularly in patients who undergo HTO.

Another study^20^ that met the inclusion criteria for the present review demonstrated results that concurred with the main findings, but it could not be included in the pooled analysis owing to the use of graphs, rather than numbers, to present pre- and postoperative scores. The authors performed a prospective comparative study of return to physical activity after HTO and UKA where activity was measured with the Tegner and UCLA scores preoperatively and at 3, 6, 12, and 24 months after surgery. The findings showed that the HTO group was significantly more active than the UKA group preoperatively, but the latter had a larger improvement in physical activity such that the postoperative levels reached by those undergoing UKA were not significantly different from those of their HTO counterparts.

Although previous systematic reviews have presented findings based on return to physical activity after HTO or UKA, to our knowledge none exists that compares the differences in activity levels between the procedures. Ekhtiari et al^12^ conducted a systematic review into return to work and sports after HTO and found that 85.2% of patients receiving OW HTO returned to a level of physical activity that was equal to or greater than their preoperative status. These results were reflected in the findings of the present review. Waldstein et al^54^ conducted a similar systematic review but investigated patients returning to physical activity after UKA, finding that participation in physical activity decreased up to 9% postoperatively. This is in contrast to the findings of the present review. It should, however, be noted that a decrease in sports participation does not necessarily equal a decrease in activity levels among the patients who remained active. This can be exemplified by scrutinizing the only study included in the present review that was included in the Waldstein et al review: Walker et al.^55^ Walker et al found a 2% decrease in the number of sports activities participated in postoperatively compared to the number of activities performed prior to the onset of symptoms, yet a significant pre- to postoperative increase in physical activity levels was observed according to the Tegner and UCLA scores. Based on this evidence, UKA may lead to a decrease in the number of activities participated in, but the level at which the remaining activities are performed increases.

Four meta-analyses compared outcomes of HTO and UKA, but walking velocity was the only physical activity–related outcome that they examined. Two meta-analyses^15,43^ found no significant differences between the procedures with regard to walking velocity, whereas the remaining 2 meta-analyses^14,17^ found that UKA resulted in faster postoperative velocity. The finding of Gandhi et al^15^ was criticized by its authors as being potentially underpowered, since only 2 studies in their review reported walking velocity, constituting approximately 30 HTO and 30 UKA cases. The meta-analysis by Santoso and Wu^43^ used the same studies as Fu et al^14^ to assess walking velocity but came to different conclusions. This was explained by the authors as being due to their analysis including the HTO and UKA results from 1 particular study^19^ that also involved patients who had undergone TKA. In contrast, Fu et al included the results of the TKA cohort with the UKA outcomes, thereby weakening their position. There is conflicting evidence at best regarding walking velocity after HTO and UKA. Until further research is conducted, it should not be used as a parameter for comparing the superiority of one procedure over the other with regard to postoperative physical activity.

### Strengths and Limitations

The pooled analysis conducted on the demographic and operative data, as well as the most commonly used patient-reported outcome measures, is a strength of this systematic review. The similar mean follow-up time between the pooled HTO and UKA groups allowed for a more reliable comparison of outcomes. However, the variation in operative techniques and equipment used, the low number of prospective RCTs, and the high number of retrospective or noncomparative studies contributed to the heterogeneity of the included articles and the lack of statistical analysis performed on the data. Conclusions drawn based on the pooled analysis offer only an approximate indication of the current situation regarding HTO, UKA, and postoperative physical activity.

The results of the present analysis are limited to patients who underwent HTO with internal plate fixators, since this is the most common form of fixation used.^27,41^ Alternative forms of fixation are available, including external fixators, staples, or spacer implants inserted into the osteotomy gap. Studies that included such fixation methods were not incorporated in the present review, as they could have confounded the results because of the differences in their indications and fixation technique,^7,11,24^ as well as the clinical and biomechanical outcomes that they achieve as compared with internal plate fixation.^2,4,37,59^

## Conclusion

This systematic review of the literature showed that HTO and UKA are effective procedures that allow patients to return to an equal or greater level of physical activity postoperatively as compared with their preoperative status. Patients who underwent HTO were more physically active pre- and postoperatively, but patients undergoing UKA experienced an overall greater increase in their physical activity levels. Activity after HTO may be influenced by intraoperative factors such as the implant used and the decision to include graft material in the osteotomy gap, although this requires further research. Finally, the indications for osteotomy are expanding and, despite traditional teaching, patients with a high BMI were also able to return to good levels of physical activity after HTO and UKA surgery.
